# An Analytical Perspective on Determination of Free Base Nicotine in E-Liquids

**DOI:** 10.1155/2020/6178570

**Published:** 2020-03-10

**Authors:** Vinit V. Gholap, Rodrigo S. Heyder, Leon Kosmider, Matthew S. Halquist

**Affiliations:** ^1^Department of Pharmaceutics, School of Pharmacy, Virginia Commonwealth University, Richmond 23298, VA, USA; ^2^Department of Pharmaceutics, Pharmaceutical Engineering, School of Pharmacy, Virginia Commonwealth University, Richmond 23298, VA, USA; ^3^Department of General and Inorganic Chemistry, Medical University of Silesia, Katowice FOPS in Sosnowiec, Jagiellonska 4, 41-200 Sosnowiec, Poland

## Abstract

In electronic cigarette users, nicotine delivery to lungs depends on various factors. One of the important factors is e-liquid nicotine concentration. Nicotine concentration in e-liquids ranges from 0 to >50 mg/mL. Furthermore, nicotine exists in protonated and unprotonated (“free base”) forms. The two forms are believed to affect the nicotine absorption in body. Therefore, in addition to total nicotine concentration, e-liquids should be characterized for their free base nicotine yield. Two approaches are being used for the determination of free base nicotine in e-liquids. The first is applying a dilution to e-liquids followed by two methods: Henderson–Hasselbalch theory application or a Liquid-Liquid Extraction. The second is the without-dilution approach followed by ^1^H NMR method. Here, we carried out controlled experiments using five e-liquids of different flavors using these two approaches. In the dilution approach, the Henderson–Hasselbalch method was tested using potentiometric titration. The accuracy was found to be >98% for all five e-liquid samples (*n* = 3). A Liquid-Liquid Extraction was carried out using toluene or hexane as extraction solvent. The Liquid-Liquid Extraction technique was found to be limited by solvent interactions with flavors. Solvent extractions resulted in flavor dependent inaccuracies in free base nicotine determination (5 to 277% of calculated values). The without-dilution approach was carried out using ^1^H NMR as described by Duell et al. This approach is proposed to offer an independent and alternative scale. None of the methods have established a strong correlation between pre- and postvaporization free base nicotine yield. Here we present comparative results of two approaches using analytical techniques. Such a comparison would be helpful in establishing a standardized method for free base nicotine determination of e-liquids.

## 1. Introduction

Nicotine is an alkaloid with a weakly basic nature. Based on the solvent nature, it can exist as protonated and free base nicotine. Historically, tobacco companies have been using alkaline chemical substances such as ammonia or its related basic compounds in manufacturing of cigarettes [1, 2]. Although the tobacco companies have denied the effect of such substances on the alteration of the absorption of nicotine, USFDA argued that using such alkaline substances shifts the balance from protonated (NicH^+^) to free base form (Nic) of nicotine in tobacco to cause a rapid and efficient absorption of nicotine in consumers [1, 2]. Based on the Pankow theory and studies by tobacco companies [3], free base nicotine is responsible for harshness or impact in smokers. Here, impact means “sudden, sharp but short lived sensation which is noticed immediately [when] smoke makes contact with the back of the throat” [3, 4]. The possible reason for such harshness is the free base nicotine in aerosol which is readily volatile and can deposit quickly in upper respiratory track [1, 3]. Based on pH partition hypothesis of drug absorption [5], any drug easily penetrates biological membrane barrier in an unionized form. Therefore, free base nicotine is believed to easily cross the biological membrane of the respiratory tract and lead to nicotine's rapid absorption. Many authors, based on their in vitro and in vivo studies, have confirmed that rate of nicotine absorption is higher for basic than acidic nicotine solutions and aerosols [6–10].

In contrast, PAX Labs Inc. in its patent has shown that a salt form of nicotine, which is mostly protonated nicotine, gave higher plasma nicotine concentration (*C*_max_) than free base nicotine. One possible explanation for this outcome could be that the less volatile and particulate nature of protonated nicotine can have deeper penetration into the lungs where absorption is more rapid than upper respiratory tract. Additionally, since protonated nicotine does not cause impact in throat [11], user may perform deep inhalation which can result in higher amount of nicotine reaching to alveolar region.

Experimentally it is still not clear how nicotine crosses biological membrane. With the two conflicting theories and experimental data about the absorption of free base vs. protonated nicotine, independent and unbiased scientific research is still not available to determine the “rate of nicotine uptake in smokers/vapers as a function of its free base dose”. In either case, it is important to classify e-liquids based on their free base or protonated nicotine yield for two reasons:

- Frequent high amounts of nicotine absorption can lead to its addiction. Unlike European regulations [12], FDA does not specify a cap on maximum nicotine content in e-liquids. Nicotine content in e-liquids ranges from 0 to >50 mg/mL [13]. JUUL products, which have gained high popularity in recent years, have about 50 mg/mL content of nicotine [14] which is believed to yield mainly the protonated form (NicH^+^). Additionally, the current 3rd and 4th generation box mod e-cigarette devices allow control over power settings such that high power gives high nicotine delivery. The question that arises here is whether such a high amount of nicotine in e-liquids is justifiable if protonated nicotine is suspected of higher absorption. Increased exposure of nicotine in youth can affect the prefrontal cortex development, leading to a deficiency in attention and lasting effects on cognitive functions [15]. Therefore, to control the youth addiction of nicotine, it is important to address the free base or protonated nicotine content in e-liquids for their adequate regulation.
- Unlike free base nicotine, salt based nicotine causes less harshness in throat hit and thus improves palatability and smoothness of e-cig aerosol [11]. Based on popularity surveys and flavor studies of several other e-liquids, different flavors of e-liquids of equal strength are perceived to give different nicotine impacts [16–18]. Thus, classifying e-liquids based on their free base nicotine delivery would help in understanding the acceptance criteria of e-liquids by vapers.

We have recently proposed a potential standard method for analyzing total nicotine content in e-liquids using peak purity criteria by HPLC [19]. However, there is no standardized method yet to quantify free base nicotine yield from e-liquids.

**Figure 1:**
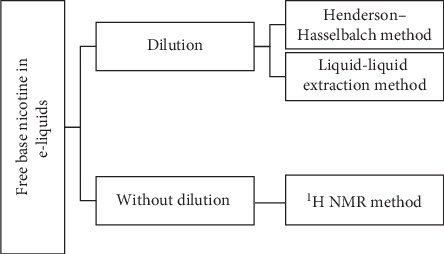
Schematic of methods for free base nicotine determination in e-liquids.

Figure 1 explains the two major approaches currently being used for the determination of free base nicotine in e-liquids. The first is a dilution approach and the second is without-dilution approach. A dilution approach has been utilized in two methods: Henderson–Hasselbalch method and Liquid-Liquid Extraction method. Without-dilution approach has been followed by ^1^H NMR method alone.

A dilution approach followed by Henderson–Hasselbalch method has been used by many authors [13, 20–22]. However, due to various concerns about the effect of flavors on the accuracy of results obtained by Henderson–Hasselbalch equation, a Liquid-Liquid Extraction method was proposed by El-Hellani et al.

It can be argued that the dilution approach causes change in e-liquids' original solvent system, which is majorly nonaqueous. Duell et al. argued that this change in solvent system may not give a true picture of free base nicotine in e-liquids. Additionally, different dilution factors might influence the results and make comparisons challenging. To address this issue, Duell at el proposed a without-dilution approach by which free base nicotine is determined using ^1^H NMR.

To compare and present critique of the two approaches, we carried out controlled experiments using five e-liquids of different flavors. We present results using various analytical techniques that will facilitate the establishment of a standardized method by regulatory bodies for classifying e-liquids based on their free base nicotine yield.

## 2. Materials and Methods

### 2.1. Instrumentation

Analysis of nicotine by potentiometric titration was carried out using a TruLab pH 1310P (YSI Incorporated, Xylem Inc, USA) potentiometric pH meter with TruLine 15 glass electrode selective to H^+^ ions and containing silver chloride reference electrode. Liquid-Liquid Extraction was carried out using a Waters Alliance 2695 quaternary pump HPLC equipped with Waters 996 PDA Detector, Hypersil Gold Phenyl column (150 mm × 4.6 mm, 3 *μ*m, Thermo Scientific™, USA), and a Security Guard Cartridge Phenyl (4 mm × 2.0 mm, Phenomenex, USA). Waters Empower 2 software was used for processing data. NMR analysis was carried out using Bruker NanoBay AVANCE III 400 MHz NMR spectrometer (Bruker Corporation, USA).

### 2.2. Chemicals and Reagents

(−)-Nicotine liquid standard (purity ≥ 99%) was purchased from Sigma Aldrich, USA. HPLC grade acetonitrile, methanol, and water were purchased from BDH Chemicals, VWR, USA. Ortho phosphoric acid (85%) was purchased from Merck, USA. Triethyl amine, tert-butyl amine, and hydrochloric acid (37%) were purchased from Sigma Aldrich, USA. Glacial acetic acid was purchased from Macron Fine Chemicals, USA. Sodium hydroxide (10N) was purchased from BDH Chemicals, VWR, USA. Propylene glycol was purchased from Amresco LLC, VWR, USA. USP grade vegetable glycerin was purchased from JT Baker, USA. e-Liquid flavors (without nicotine) of menthol, tobacco, fruit, sweet, and coffee were purchased from Direct Vapor online vape shop, USA. NMR analysis was carried out using coaxial inserts for 5 mm NMR precision sample tube (WGS-5BL-SP, Wilmad-LabGlass, USA). Dimethyl sulfoxide-d6 was purchased from Sigma Aldrich, USA.

### 2.3. Preparation of Reagents

e-Liquids were prepared by dissolving liquid nicotine standard in each flavor. Similarly, quality control (QC) samples were prepared by dissolving liquid nicotine standard in unflavored matrix of propylene glycol and vegetable glycerin (1 : 1, v/v). Standards, mobile phase, and diluent for HPLC analysis were prepared as described earlier [19]. In potentiometric titration experiments, pH adjustment and titration were carried out using 0.1N NaOH and 0.1N HCl. Water used for all preparations was HPLC grade purified water. Sample preparation for NMR analysis was carried out based on the method suggested by Duell et al. [23].

### 2.4. Dilution Approach

#### 2.4.1. Henderson–Hasselbalch Method

Henderson–Hasselbalch method for determination of free base nicotine yield of e-liquids was tested using potentiometric titration.

*(1) Potentiometric Titration*. Potentiometric titration is a technique based on measuring the change in potential after addition of titrant to a solution containing counterions to that of the titrant. Equivalence point is the point at which the rate of change in potential per each incremental addition of titrant is maximum. Change in potential was measured for each incremental addition of titrant, 0.1N NaOH.

- Determination of pKa of pyrrolidine group of nicotine:
- Nicotine standard solution of 0.8 mg/mL was prepared in water at three different pHs: 7, 8, and 9. The pH adjustments were carried out using 0.1N HCl and 0.1N NaOH. Potentiometric titration of these solutions was carried out against 0.1N NaOH. Equivalence point was determined by a first derivative plot of change in potential vs. volume of NaOH added. pKa was calculated using Henderson–Hasselbalch equation [5]:

(1) $$\begin{array}{c} \text{pH}=\text{pKa}+\log\frac{\left[\text{Nic}\right]}{\left[{\text{NicH}}^{+}\right]}, \end{array}$$

where Ka is the nicotine's acid dissociation constant, [Nic] is the free base nicotine concentration, and [NicH^+^] is the protonated nicotine concentration.

**Figure 2:**
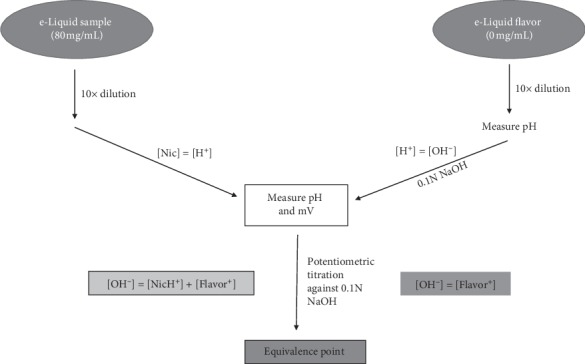
Schematic of methodology of potentiometric titration to measure protonated nicotine [NicH^+^].

(b) Determination of free base nicotine from e-liquids
  Potentiometric titration was carried out to quantify NicH^+^ in e-liquid solutions (10x diluted). e-Liquids containing 80 mg/mL of nicotine were prepared using five different flavors: menthol, tobacco, fruit, sweet, and coffee. A control e-liquid was prepared in PG : VG (1 : 1, v/v). Using the dilution approach, each e-liquid was diluted 10x in water to achieve final nicotine concentration of 8 mg/mL. Due to sensitivity of the potentiometer and for ease of the titration, nicotine concentration of diluted samples was set as 8 mg/mL. The pH of the diluted e-liquids was measured and potentiometric titration was carried out against 0.1N NaOH. Similarly, placebo flavors and placebo control, all without nicotine, were analyzed after by 10x dilution in water. First, the pH of each diluted placebo was measured. Subsequently, the pH of each diluted placebo was adjusted to the pH of their respective diluted e-liquid sample using 0.1N NaOH. Finally, potentiometric titration was carried out similar to that of samples. Schematic representation of the methodology is described in Figure 2. Flavor cations generated, if any, after pH adjustment of placebo by 0.1N NaOH are subtracted from total titrated cations generated from sample to give the NicH^+^. The percentage (%) of free base nicotine (Nic) was calculated using equations (2)–(4).

Since both the sample and placebo were diluted in the same grade of water (HPLC grade with constant pH), effect of dissolved CO_2_ in water was nullified and was not considered for calculations:

(2) $$\begin{array}{c} \left[{\text{NicH}}^{+}\right]=\left[\text{total cations}\right]-\left[\text{flavor cations}\right], \end{array}$$

(3) $$\begin{array}{c} \left[\text{Nic}\right]=\left[\text{Nic}+{\text{NicH}}^{+}\right]-\left[{\text{NicH}}^{+}\right], \end{array}$$

(4) $$\begin{array}{c} \%\text{ Nic}=\frac{\left[\text{Nic}\right]}{\left[\text{Nic}+{\text{NicH}}^{+}\right]}\times 100. \end{array}$$

#### 2.4.2. Liquid-Liquid Extraction Method

**Figure 3:**
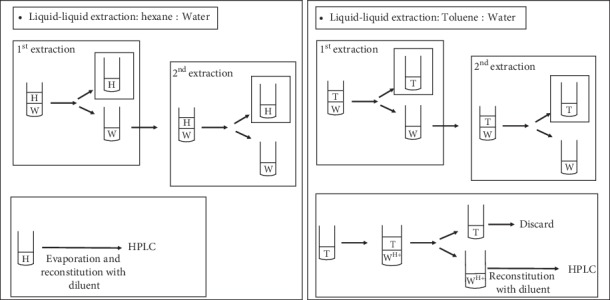
Schematic of methodology of measurement of free base and protonated nicotine by extraction using hexane (H) or toluene (T) in water (W) or acid (W^H+^).

Based on the hypothesis proposed by El-Hellani et al. [24], organic solvents should demonstrate selective extraction of free base nicotine from an aqueous solution of nicotine. Therefore, the extraction method was developed based on the earlier research published by El-Hellani et al. [24]. Briefly, nicotine standard solutions (0.8 mg/mL) of five different pHs, namely, 5, 7, 9, 11, and 13, were prepared in water. El-Hellani et al. have used toluene as extracting solvent. To assess the extraction efficiency of toluene, we compared it with another well studied organic solvent, hexane, which had been used for recovery of nicotine from tobacco extracts [25]. Extraction efficiency was determined for single and double extractions for each solvent. Nicotine in aqueous and organic layer was quantified by an HPLC method [19]. A similar procedure was followed for nicotine extraction using e-liquids (8 mg/mL) of five different flavors, namely, menthol, tobacco, fruit, sweet, and coffee. Extraction method involves 10x dilution of e-liquids in aqueous layer. Thus, final concentration of e-liquids achieved in aqueous layer was similar to standards, i.e., 0.8 mg/mL of nicotine. Additionally, pH measurement was carried out for both standards and samples (10x diluted) to calculate % free base using Henderson–Hasselbalch equation.  Figure 3 describes the methodology of extraction.

### 2.5. Without-Dilution Approach

#### 2.5.1. ^1^H NMR Spectroscopy

Based on methodology by Duell et al. [23], free base nicotine in e-liquids was determined by ^1^H NMR spectroscopy. Briefly, e-liquids were prepared at concentration of 8 mg/mL using the same five different flavors (menthol, tobacco, fruit, sweet, and coffee). The control e-liquid was prepared in propylene glycol : vegetable glycerin (PG : VG, 54 : 46, v/v). Free base and protonated standards were prepared by combining the aliquots of the control sample with base (t-butylamine : nicotine, 1 : 1 mol : mol) and with acid (acetic acid : nicotine, 5 : 1, mol : mol).

NMR spectroscopy was carried out using precision coaxial NMR inserts with experimental parameters as described by Duell et al. [23]. Free base nicotine was calculated using equations (5) and (6) [23] and (7), based on difference in chemical shifts between aromatic hydrogens and hydrogens of the methyl (-CH_3_) group which connects to protonable nitrogen (N) atom of the pyrrolidine ring:

(5) $$\begin{array}{c} \Delta \delta =\left[\delta \text{H aromatic proton}\left(\text{i.e.},\text{Ha through Hd}\right)\right]-\left[\delta \text{He}\right], \end{array}$$

(6) $$\begin{array}{c} \alpha fb=\frac{\left[\left(\Delta \delta \text{ commercial sample}\right)-\left(\Delta \delta \text{ monoprotonated standard}\right)\right]}{\left[\left(\Delta \delta \text{ free base standard}\right)-\left(\Delta \delta \text{ monoprotonated standard}\right)\right]}, \end{array}$$

(7) $$\begin{array}{c} \%\text{ Nic}=\alpha fb\times 100. \end{array}$$

## 3. Results

### 3.1. Dilution Approach

#### 3.1.1. Henderson–Hasselbalch Method

*(1) Potentiometric Titration*.

**Figure 4:**
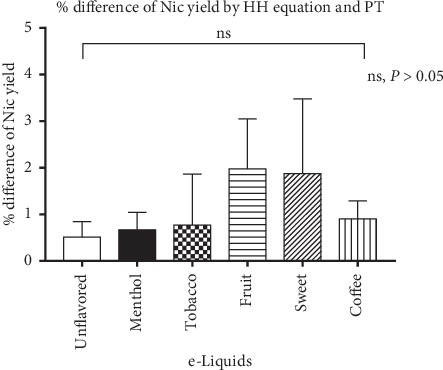
Percentage difference of free base nicotine (Nic) in e-liquids by HH equation vs. potentiometry (ns, *P* > 0.05; statistical analysis was carried out using GraphPad Prism 7.0).

- Determination of pKa of pyrrolidine group of nicotine:
- To confirm the accuracy of potentiometric titration, the pKa was determined using a nicotine standard solution (0.8 mg/mL) at pHs 7, 8, and 9. Each solution was titrated against 0.1N NaOH, and equivalence point was determined by a first derivative plot of change in potential vs. volume of NaOH added. Using equation (1), the pKa of pyrrolidine group was found to be 8.17 ± 0.12 at 19.0 ± 1°C.
- Determination of free base nicotine from e-liquids:
- Using potentiometric titration, e-liquids were analyzed for % free base nicotine yield as described in (b) in Section 2.4.1 and Figure 2. Using equations (2)–(4), free base nicotine was calculated and compared with the theoretical results obtained from Henderson–Hasselbalch equation. The mean percentage difference between experimental and theoretical results (HH-PT) was found to be 1.12 ± 0.64 for all e-liquids (Table 1 and Figure 4).

#### 3.1.2. Liquid-Liquid Extraction Method

**Figure 5:**
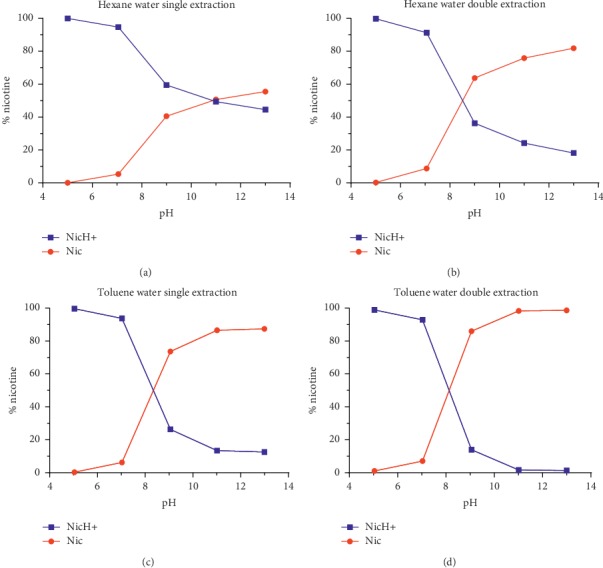
Extraction of free base (Nic) and protonated nicotine (NicH^+^) by (a) hexane-water single extraction, (b) hexane-water double extraction, (c) toluene-water single extraction, and (d) toluene-water double extraction. Note. Percent recovery of hexane and toluene extractions is 98.04 ± 1.66 and 97.10 ± 3.47, respectively. All results are in triplicate with RSD < 2.0%.

As discussed in the methodology, free base nicotine quantification was carried out using hexane and toluene to selectively extract free base nicotine from aqueous solutions at pH 5, 7, 9, 11, and 13.  Figure 5 describes the hexane and toluene extraction efficiency for nicotine standard solutions at various pHs. Since nicotine is expected to follow Henderson–Hasselbalch equation in its standard solution in water, free base nicotine extraction results were compared with the theoretical values obtained from the equation.

Based on the % difference between the theoretical values and experimental results, toluene was found to have higher extraction efficiency (96.32 ± 3.51%) for free base nicotine as compared to hexane (77.34 ± 3.72%) (Tables 2 and 3 and Figure 5).

We also calculated nicotine's partition coefficient in toluene-water system (*K*_T/W_) as 6.92 after a single extraction. In contrast, the partition coefficient of nicotine in hexane-water system (*K*_H/W_) was found to be 1.25. Therefore, based on the results of nicotine standard solutions, toluene was used as an extracting solvent for e-liquids. e-Liquids of five different flavors, namely, menthol, tobacco, fruit, sweet, and coffee, were used for toluene extraction as described in Section 2.4.

Since the pKa of any molecule depends on the solvent it is dissolved in [26], the pKa of nicotine can be perturbed by a solvent environment due to presence of flavoring chemicals, PG or VG. However, it was confirmed from the potentiometric experiment (Section 2.4.1 and Figure 4) that 10x dilution of e-liquids in water smooths out the effect of other chemicals on pKa of nicotine in water.

**Figure 6:**
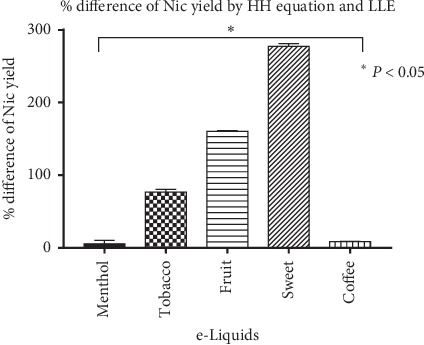
Percentage difference of free base nicotine (Nic) yield of e-liquid samples by HH equation vs. LLE (toluene-water extraction) (^*∗*^*P* < 0.05; statistical analysis was carried out using GraphPad Prism 7.0).

Therefore, similar to the standard solution, Henderson–Hasselbalch equation was used to calculate the free base nicotine yield from e-liquid samples. The mean percentage difference between theoretical results and experimental results (HH-LLE) was found to be variable ranging from 5.74 to 277.02% for five e-liquids (Table 4 and Figure 6).

### 3.2. Without-Dilution Approach

#### 3.2.1. ^1^H NMR Spectroscopy

As described in Section 2.5.1, the ^1^H NMR experiment was performed on e-liquids without dilution. Using equations (5)–(7), the % free base nicotine was calculated for five e-liquid flavors (Table 5). As the e-liquids were not diluted in water, the results were found to be different from the results obtained by Henderson–Hasselbalch method (Table 6) with a % deviation from 3.70 to 26.93%.

## 4. Discussion

Considering the addictive nature of nicotine, e-liquids need to be regulated for the total nicotine content as well as free base nicotine yield. There are published methods for determining total nicotine content in e-liquids using gas chromatography and liquid chromatography coupled with mass detectors [27–29]. Recently, we have proposed a HPLC method based on peak purity criteria for accurate quantification of total nicotine content [19]. In contrast, methods for quantification of free base nicotine in e-liquids are still being debated for their accuracy.

### 4.1. Dilution Approach

#### 4.1.1. Henderson–Hasselbalch Method

Many authors have used this approach by diluting e-liquids in fixed amount of water followed by pH measurement [13, 20–22]. This dilution method has been used for analytical characterization of smokeless tobacco [30]. However, e-liquids are complex formulations with variety of flavoring chemicals. Considering the autoprotolysis constant theory, pKa value of any molecule depends upon solvent's acid/base properties and polarity [26]. The use of Henderson–Hasselbalch method by pH measurement of diluted e-liquids and calculation of free base nicotine yield raises concerns about the effect of flavoring chemicals, PG and VG, on pKa of nicotine in water. Therefore, using a fixed dilution factor (10x), we tested the Henderson–Hasselbalch method using potentiometry.

The accuracy of the potentiometric titration was confirmed by calculating pKa of the pyrrolidine group of nicotine as described in (a) in Section 3.1.1. Gonzalez et al. [31] had extensively studied pKa of nicotine against temperature and reported the value of pyrrolidine pKa as 8.20 and 8.06 at 15°C and 20°C, respectively. Based on our results, the pKa of nicotine was found to be 8.17 ± 0.12 at 19°C ± 1, which is close to the value reported by González and Monge [31].

We carried out potentiometric study to test Henderson–Hasselbalch method and we used 80 mg/mL concentration of nicotine. The reason for using high concentration of nicotine was to overcome the low sensitivity of the potentiometer and for ease of titration.

Based on results described in (b) in Section 3.1.1, the mean percentage difference between potentiometric titration and Henderson–Hasselbalch method results for each sample was <2.0%. Similarity between these two methods is uniform across all flavors (Kruskal–Wallis Test, *P* > 0.05) (Figure 4). Thus, pKa of nicotine is not found to be perturbed by flavoring chemicals, PG or VG, after 10x dilution in water. In other words, 10x dilution smooths out the effect of flavoring chemicals, PG or VG, on pKa of nicotine.

Since, pKa of a molecule is independent of its concentration in a particular solvent, the dilution approach using Henderson–Hasselbalch method can also be applied to e-liquids of lower nicotine concentrations for quantifying free base nicotine yield.

#### 4.1.2. Liquid-Liquid Extraction Method

Under the dilution approach, El-Hellani et al. proposed alternative method which was Liquid-Liquid Extraction. The method proposed by El-Hellani et al. [24] is based on their hypothesis that “free base nicotine is selectively extracted in organic solvent, toluene.” We performed a detailed study of this method with additional parameters such as partition coefficients and extraction efficiency.

It is well established fact that the protonated from of nicotine always remains in aqueous phase [32]. This fact has been reconfirmed by our study (Figure 5) where almost 100% protonated nicotine in a standard solution of pH 5 remained in aqueous phase during extraction phase. As nicotine starts to exist in protonated as well as free base forms at pH > 5, the distribution of nicotine in aqueous and organic phase becomes much more complex and is primarily governed by the partition theory of nicotine in aqueous and organic solvents [32]. Partition coefficient is the ratio of unionized molecules of a compound in two immiscible liquids [5]. Based on our extraction study (Section 3.1.2), we are reporting nicotine's partition coefficient in toluene (*K*_T/W_) as 6.92 which is higher than its partition coefficient in hexane (*K*_H/W_), 1.25. Thus, extraction efficiency of toluene is about 87% higher than hexane. Therefore, after a second extraction, toluene shows almost 98% extraction efficiency for free base nicotine.

Nicotine is a weak base with two nitrogen atoms capable of accepting protons. Out of the two rings, pyrrolidine ring (pKa 8.20) is more basic than the pyridine ring (pKa 3.41) [32]. Based on the pKa values and Henderson–Hasselbalch theory, the pyridine ring ionizes at lower pH (<5.0) as compared to pyrrolidine ring. Thus, the contribution of pyridine ring towards ionization of nicotine can be neglected at higher pH. Based on the ionization theory of weak acids and weak bases [5], nicotine in aqueous solution at different pH (5 ≥ pH ≥ 13) should ionize as per Henderson–Hasselbalch theory. Considering toluene's extraction efficiency, the % free base nicotine obtained after toluene double extraction should also match the theoretical values obtained from Henderson–Hasselbalch equation. This hypothesis has been confirmed from data obtained by El-Hellani et al. [24] and our results are described in Figure 5. After confirming the toluene's extraction efficiency using nicotine standard solutions at different pHs, toluene was chosen as extracting solvent for determining free base nicotine yield of e-liquids. Toluene double extraction was performed on e-liquids with five distinct flavors. Extraction method involves 10x dilution of e-liquids in aqueous layer. Therefore, as described in Sections 2.4.2 and 3.1.2, pH of e-liquid samples (10x diluted) was also measured and % Nic was calculated using Henderson–Hasselbalch equation. After extraction, the mean percentage difference between experimental and theoretical free base nicotine yield (HH-LLE) was found to be significantly variable (5.74 to 277.02%, Table 4 and Figure 6). The possible reason for such high variation could be that flavors in e-liquid samples interact with toluene and affect toluene's extraction efficiency for nicotine. Variation between Liquid-Liquid Extraction and Henderson–Hasselbalch method values can be found across all flavors (Kruskal–Wallis Test, *P* < 0.05).

In addition, e-liquids diluted in water can be a mixture of weak acids or bases. According to Le Chatelier's principle, after single extraction of Nic in toluene, there can be a change in equilibrium between Nic and NicH^+^ before the second extraction. Thus, values in Liquid-Liquid Extraction can be overestimated. Therefore, it can be concluded that, under dilution approach, Liquid-Liquid Extraction is not an accurate method for determining free base nicotine yield of e-liquids.

#### 4.1.3. Critique of Dilution Approach

- Using the dilution approach, only Henderson–Hasselbalch method based on a fixed dilution was found to be accurate and not the Liquid-Liquid Extraction.
- The dilution approach is a simple way to determine free base nicotine yield of e-liquids.
- The major critique of the dilution approach is that dilution changes the solvent environment of e-liquids which is mostly nonaqueous liquids. However, it can be argued that the purpose of the dilution approach is to provide a pH relevant scale for classifying e-liquids based on their free base nicotine yields. To estimate the accuracy and relevance of the scale to postvaporization exposure of nicotine, it is important to establish pre- and postvaporization correlation. Such correlation can be established if stable medium is used for pre- and postvaporization analysis. Aerosol is a highly unstable phase to analyze as it is for free base nicotine. Dilution approach provides a stable “medium” for collecting aerosol for analysis.
- The ratio in which postvaporized aerosol interacts with lung surface fluid cannot be determined. Thus, the dilution factor, e.g., 10x in current study, is just an arbitrary number. However, such dilution factor should be studied for effect of solvent environment on pKa of nicotine. Based on our results, we have shown that pKa of nicotine in water is not perturbed by solvent environment of flavoring chemicals, PG or VG, after a 10x dilution factor. Therefore, we would like to propose 10x dilution factor in following a dilution approach.
- This scale would be uniform across all studies only if the dilution factor and water grade (for consideration of dissolved CO_2_) are fixed.
- As descried earlier, using dilution approach, it is necessary to establish a correlation between pre- and postvaporization free base nicotine yield. Therefore, additional research is required.

### 4.2. Without-Dilution Approach

Based on the abovementioned critique of the dilution approach, Duell et al. have argued that water should not be considered while measuring protonated and free base nicotine in e-liquids. The authors have stressed on measuring free base nicotine from e-liquids without dilution for measuring free base nicotine.

#### 4.2.1. ^1^H NMR Spectroscopy

Duell et al. [23] proposed without-dilution approach and used ^1^H NMR spectroscopy to quantify free base nicotine in e-liquids. The method is based on quantifying the difference between chemical shifts of -CH_3_ group connected to protonable N atom of the pyrrolidine ring as described in Section 3.2.1. We replicated the procedure to compare the results of the same five e-liquids used earlier in the extraction and potentiometry study. Based on the results described in Table 5, the percentage of free base nicotine in the five e-liquids gives different values from the results obtained from Henderson–Hasselbalch method (Table 6) with a percentage difference ranging from 3.70 to 26.93%.

#### 4.2.2. Critique of Without-Dilution Approach

- Without-dilution approach measures e-liquids in their original nonaqueous state. If the method is considered to be accurate, the ^1^H NMR method can provide free base nicotine determination of e-liquids in its original state (without dilution). However, there are some concerns for the method to be considered as accurate.
- Selectivity and resolution: Since e-liquids are a mixture of several chemicals in different concentrations and ^1^H NMR detects all hydrogens from these molecules, there is a possibility of “-CH_3_ peak region” overlap of nicotine and other flavoring chemicals. In some of the e-liquid samples analyzed, NMR method was found to be limited by selectivity and resolution of nicotine's -CH_3_ peak. For a better identification of the peaks relative to nicotine, two-dimensional NMR (2D-NMR), such as HSQC/HMQC, could be applied. However, as the sample has several components with many unknowns, this approach would be complex and may not be enough for this identification in variety of e-liquids when peaks are overlapped/distorted.
- Limit of detection: In case of ^1^H NMR, the peaks are recorded as relative intensities compared to the highest intensity peak. In the case of high concentrations of flavoring chemicals and a low concentration of nicotine in e-liquids, nicotine's -CH_3_ peak can merge in noisy baseline or other low intensity peaks. “-CH_3_” peak for nicotine was not detected for samples with nicotine concentration <3.0 mg/mL. This compromise with the limit of detection has not been addressed yet.
- Baseline: As a mixture of several compounds and high ratio of PG : VG to nicotine, some e-liquids have shown a drift in a baseline leading to incorrect integration of the peaks with small intensity. Such incorrect integration can cause incorrect identification of nicotine's -CH_3_ peak.
- Another challenge of without-dilution approach is that it does not provide a medium to collect aerosol. To estimate the accuracy and relevance of without-dilution approach to postvaporization exposure of nicotine, it is necessary to establish a correlation between pre- and postvaporization free base nicotine yield. Earlier research has analyzed postvaporized aerosol by collecting it in NMR sample tube [23]. Postvaporized aerosol is highly unstable phase to analyze free base nicotine in aerosol form. Such analysis can lead to overestimation or underestimation of free base nicotine. The data generated is too limited to address this concern [23].

## 5. Conclusions

Currently there are two approaches being used for determination of free base nicotine in e-liquids. The first is dilution approach and the second is without-dilution approach. Each method has some advantages and shortcomings.

Dilution approach provides a pH relevant scale for classifying e-liquids based on their free base nicotine yields. Using dilution approach, Henderson–Hasselbalch method is found to be more accurate than the Liquid-Liquid Extraction method. For dilution approach to be uniform across all studies, dilution factor and water grade need to be fixed. Based on our study, we would like to propose 10x dilution factor.

Although the dilution approach is being used by several researchers, some authors have argued about the change in solvent system. To address this issue, without-dilution approach has been proposed to measure free base nicotine in e-liquids without changing solvent environment. The data so far generated using this approach is too limited to address some critical concerns such as overlap of nicotine's -CH_3_ peak region with that of flavoring chemicals, selectivity, resolution, limit of detection, baseline drift, and lack of alternate method to overcome these concerns.

Both of the approaches need to establish a strong correlation between free base nicotine yield of pre- and postvaporized e-liquids. The actual delivery of free base nicotine to vapers is highly dependent on vaping profile. Therefore, it is equally important to consider variables such as battery voltage, coil temperature, puff duration, puff frequency, puff volume, and relative humidity while carrying out such a study.

Data generated from such correlation would eventually help in selecting appropriate approach for determination of free base nicotine in e-liquids and regulating the e-liquids in market.

## Figures and Tables

**Table 1:** Determination of free base nicotine in e-liquids by potentiometric titration (Henderson–Hasselbalch method).

e-Liquid flavor (80 mg/mL)	e-Liquid category	pH of 10x dilution in water	% free base nicotine by Henderson–Hasselbalch equation	Experimental % free base nicotine	% difference	Mean % difference	Std. dev.
Unflavored	PG : VG	10.22	99.12	100.00	0.88	0.52	0.33
		10.20	99.07	99.47	0.41		
		10.15	98.97	99.21	0.25		

Maui menthol	Menthol	9.48	95.31	94.42	0.93	0.67	0.37
		9.41	94.52	94.75	0.24		
		9.15	90.46	91.21	0.83		

Element tobacco	Tobacco	8.90	84.24	82.53	2.03	0.77	1.09
		8.84	82.39	82.31	0.09		
		8.82	81.85	81.69	0.19		

Motley brew	Fruit	8.89	83.96	81.61	2.80	1.97	1.08
		8.87	83.43	81.46	2.36		
		8.84	82.39	81.76	0.76		

Milkman milky cloud	Sweet	8.87	83.43	80.58	3.42	1.87	1.61
		8.72	78.01	77.85	0.21		
		8.72	78.05	76.50	1.99		

Hazelnut coffee	Coffee	9.63	96.68	95.38	1.34	0.90	0.38
		9.55	96.01	95.28	0.76		
		9.54	95.89	95.30	0.61		

					Avg.	1.12 ± 0.64	

**Table 2:** Determination of free base nicotine in standard solutions by hexane-water double extraction.

	pH	% free base nicotine by Henderson–Hasselbalch equation	% experimental free base nicotine	% difference	Mean % difference	Std. dev.
Nicotine std (0.8 mg/mL)	5.01	0.07	0.33	NA	NA	NA
			0.26	NA		
			0.25	NA		
	7.06	7.22	8.81	22.01	21.37	0.71
			8.77	21.50		
			8.71	20.59		
	9.00	87.14	63.33	27.32	26.92	0.35
			63.88	26.70		
			63.83	26.75		
	11.00	99.85	75.20	24.69	24.12	0.59
			76.37	23.52		
			75.74	24.15		
	13.00	100.00	81.08	18.92	18.23	0.63
			81.90	18.10		
			82.32	17.68		
				Avg.	22.66 ± 3.72	

Note. Extraction efficiency was calculated as “100 – mean % difference.” (77.34 ± 3.72%). NA: not applicable, since the % free base nicotine values are <1.

**Table 3:** Determination of free base nicotine in standard solutions by toluene-water double extraction.

	pH	% free base nicotine by Henderson–Hasselbalch equation	% experimental free base nicotine	% difference	Mean % difference	Std. dev.
Nicotine std (0.8 mg/mL)	5.04	0.07	1.27	NA	NA	NA
			0.97	NA		
			1.12	NA		
	7.03	6.76	6.43	4.83	8.85	4.42
			7.67	13.58		
			7.30	8.13		
	9.06	88.56	86.16	2.71	2.90	0.19
			86.01	2.88		
			85.82	3.10		
	11.02	99.86	98.30	1.56	1.61	0.07
			98.18	1.68		
			98.28	1.58		
	13.00	100.00	98.64	1.36	1.39	0.02
			98.59	1.41		
			98.61	1.39		
				Avg.	3.68 ± 3.51	

*Note*. Extraction efficiency was calculated as “100 – mean % difference.” (96.32 ± 3.51%). NA: not applicable, since the % free base nicotine values are <1.

**Table 4:** Determination of free base nicotine in e-liquid samples by toluene-water double extraction.

e-Liquid flavor	e-Liquid category	pH of 10x dilution in water (0.8 mg/mL)	% free base nicotine by Henderson–Hasselbalch equation	Experimental % free base nicotine	% difference	Mean % difference	Std. dev.
Maui menthol	Menthol	8.51	68.68	65.00	5.36	5.74	4.58
				67.74	1.37		
				61.47	10.50		

Element tobacco	Tobacco	8.01	41.00	73.25	78.64	76.90	3.48
				73.46	79.16		
				70.89	72.89		

Motley brew	Fruit	7.82	30.93	80.71	160.98	160.28	1.04
				80.65	160.78		
				80.12	159.08		

Milkman milky cloud	Sweet	7.57	19.93	76.02	281.48	277.02	3.89
				74.61	274.38		
				74.77	275.18		

Hazelnut coffee	Coffee	8.82	81.81	88.79	8.53	8.76	0.20
				89.04	8.84		
				89.10	8.91		

**Table 5:** Determination of free base nicotine in e-liquids by ^1^H NMR spectroscopy (e-liquids without dilution).

e-Liquid flavor (80 mg/mL)	e-Liquid category	% experimental free base nicotine	Mean % free base nicotine	Standard deviation
Maui menthol	Menthol	71.56	69.41	1.87
		68.34		
		68.32		

Element tobacco	Tobacco	74.83	73.60	1.07
		73.01		
		72.95		

Motley brew	Fruit	75.87	74.40	1.27
		73.64		
		73.70		

Milkman milky cloud	Sweet	75.79	75.80	0.02
		75.81		
		75.79		

Hazelnut coffee	Coffee	91.76	92.88	0.97
		93.48		
		93.40		

**Table 6:** Comparison between free base nicotine in e-liquids by dilution approach (HH) and by without-dilution approach (^1^H NMR) (e-liquids without dilution).

e-Liquid flavor (80 mg/mL)	e-Liquid category	Mean % free base nicotine by Henderson–Hasselbalch equation (with dilution)	Mean % free base nicotine by ^1^H NMR (without dilution)	% deviation
Maui menthol	Menthol	94.99	69.41	26.93
Element tobacco	Tobacco	83.3	73.6	11.64
Motley brew	Fruit	83.01	74.4	10.37
Milkman milky cloud	Sweet	82.45	75.8	8.07
Hazelnut coffee	Coffee	96.45	92.88	3.70

## Data Availability

The data used to support the findings of this study are available from the corresponding author upon request.

## Abbreviations

NA: Not applicable
LLE: Liquid-liquid extraction
PT: Potentiometric titration
HH: Henderson–Hasselbalch
Nic: Free base nicotine
NicH^+^: Protonated nicotine
e-cig: Electronic cigarette
PG: Propylene glycol
VG: Vegetable glycerine
ns: Not significant.
